# Supplementary material for: Elucidating T Cell Activation-Dependent Mechanisms for Bifurcation of Regulatory and Effector T Cell Differentiation by Multidimensional and Single-Cell Analysis
Source: Front Immunol. 2018 Jul 10;9:1444. doi: 10.3389/fimmu.2018.01444 (PMC6048294; doi:10.3389/fimmu.2018.01444)

Supplementary Figure Legends

Supplementary Figure 1. Heatmap analysis of differentially expressed genes.

A moderated t-test analysis of gene lists was applied to identify Treg-specific and Tmem-specific genes, T cell activation signature genes, and Foxp3-transduction effects genes (false discovery rate (FDR) < 0.05).

Supplementary Figure 2. SC4A analysis for choosing explanatory variables.

Top-ranked genes for each differentiation/activation process identified by combinatorial CCA implemented in the SC4A method.

Supplementary Figure 3. Principal component Analysis (PCA) and t-Distributed Stochastic Neighbor Embedding (t-SNE) analysis of the melanoma dataset.

(i) PCA was applied to the dataset using the standard method. (ii) t-SNE was applied using perplexity=30. See legends for cell groups.

Supplementary Figure 1

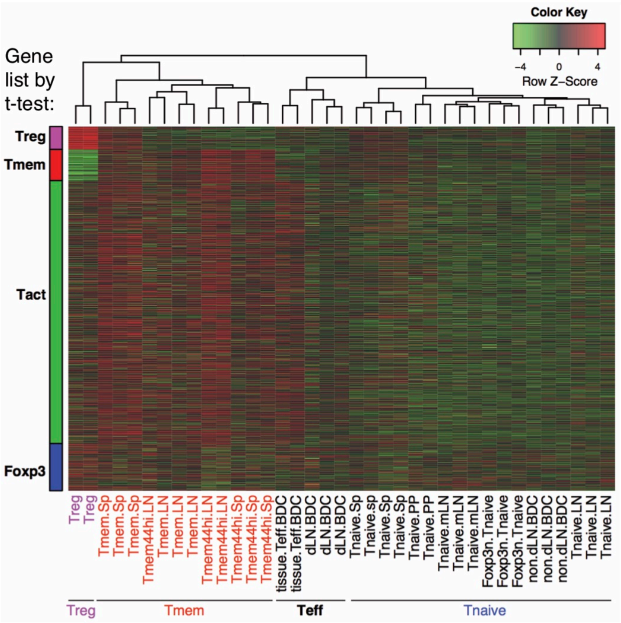

Supplementry Figure 2

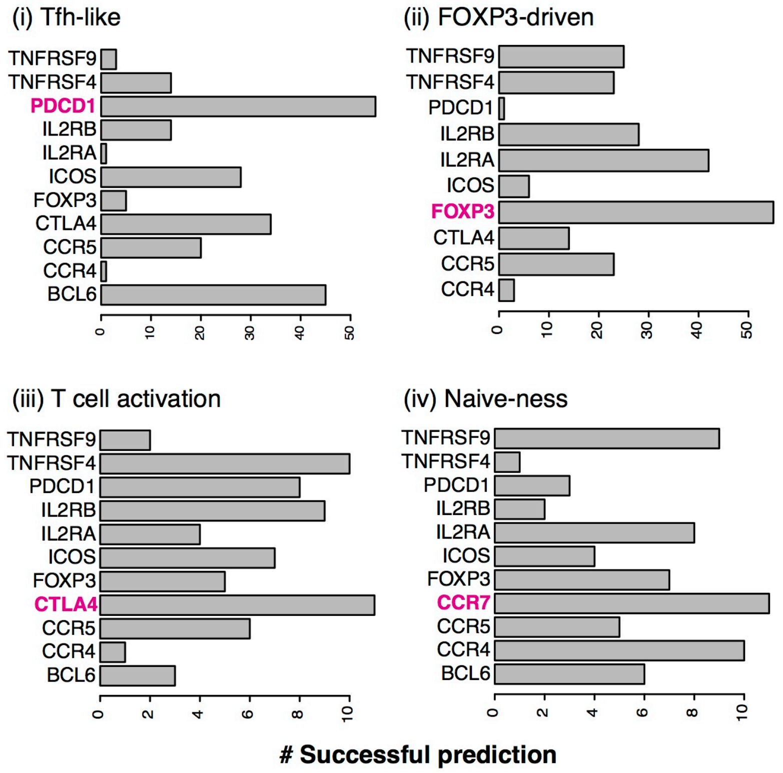

Supplementry Figure 3

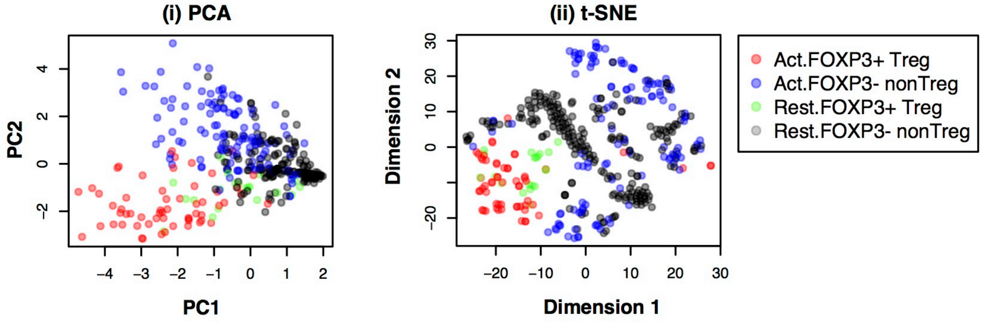

Supplement: Supplementary file 1 [file image_1.PDF]
